# Cell type-specific deletion in mice reveals roles for PAS kinase in insulin and glucagon production

**DOI:** 10.1007/s00125-016-4025-1

**Published:** 2016-06-24

**Authors:** Francesca Semplici, Angeles Mondragon, Benedict Macintyre, Katja Madeyski-Bengston, Anette Persson-Kry, Sara Barr, Anna Ramne, Anna Marley, James McGinty, Paul French, Helen Soedling, Ryohsuke Yokosuka, Julien Gaitan, Jochen Lang, Stephanie Migrenne-Li, Erwann Philippe, Pedro L. Herrera, Christophe Magnan, Gabriela da Silva Xavier, Guy A. Rutter

**Affiliations:** 1 Section of Cell Biology and Functional Genomics, Division of Diabetes, Endocrinology and Metabolism, Department of Medicine, Imperial College London, Imperial Centre for Translational and Experimental Medicine, Hammersmith Hospital, du Cane Road, London, W12 0NN UK; 2 AstraZeneca R&D, DECS, AstraZeneca R&D, Mölndal, Sweden; 3 AstraZeneca R&D, HC3020, AstraZeneca R&D, Mölndal, Sweden; 4 AstraZeneca R&D, Alderley Edge, UK; 5 Photonics Group, Department of Physics, Imperial College London, London, UK; 6 Université de Bordeaux, Institut de Chimie et Biologie des Membranes et des Nano-objets, CNRS UMR 5248, Pessac, France; 7 Paris Diderot University, Unit of Functional and Adaptive Biology (BFA), CNRS UMR 8251, Paris, France; 8 Department of Genetic Medicine and Development, Faculty of Medicine, University of Geneva, Geneva, Switzerland

**Keywords:** Basic science, Beta cell signal transduction, Islets, Mouse, PAS kinase

## Abstract

**Aims/hypothesis:**

Per-Arnt-Sim kinase (PASK) is a nutrient-regulated domain-containing protein kinase previously implicated in the control of insulin gene expression and glucagon secretion. Here, we explore the roles of PASK in the control of islet hormone release, by generating mice with selective deletion of the *Pask* gene in pancreatic beta or alpha cells.

**Methods:**

Floxed alleles of *Pask* were produced by homologous recombination and animals bred with mice bearing beta (*Ins1*^Cre^; *Pask*BKO) or alpha (*Ppg*^Cre^ [also known as *Gcg*]; *Pask*AKO) cell-selective *Cre* recombinase alleles. Glucose homeostasis and hormone secretion in vivo and in vitro, gene expression and islet cell mass were measured using standard techniques.

**Results:**

*Ins1*^Cre^-based recombination led to efficient beta cell-targeted deletion of *Pask*. Beta cell mass was reduced by 36.5% (*p* < 0.05) compared with controls in *Pask*BKO mice, as well as in global *Pask*-null mice (38%, *p* < 0.05). *Pask*BKO mice displayed normal body weight and fasting glycaemia, but slightly impaired glucose tolerance, and beta cell proliferation, after maintenance on a high-fat diet. Whilst glucose tolerance was unaffected in *Pask*AKO mice, glucose infusion rates were increased, and glucagon secretion tended to be lower, during hypoglycaemic clamps. Although alpha cell mass was increased (21.9%, *p* < 0.05), glucagon release at low glucose was impaired (*p* < 0.05) in *Pask*AKO islets.

**Conclusions/interpretation:**

The findings demonstrate cell-autonomous roles for PASK in the control of pancreatic endocrine hormone secretion. Differences between the glycaemic phenotype of global vs cell type-specific null mice suggest important roles for tissue interactions in the control of glycaemia by PASK.

**Electronic supplementary material:**

The online version of this article (doi:10.1007/s00125-016-4025-1) contains peer-reviewed but unedited supplementary material, which is available to authorised users.

## Introduction

Type 2 diabetes affects approximately one in 12 of the adult population [1] and usually involves changes in both insulin secretion [2] and insulin action [3]. Acting on the healthy pancreatic beta cell, elevated blood glucose concentrations lead to increased glucose flux across the plasma membrane, mediated by the transporter GLUT2 (plus GLUT1 in humans) [4], glucose phosphorylation catalysed by glucokinase, and enhanced glycolytic flux to fuel the citrate cycle [5]. Increased mitochondrial metabolism [6], ATP synthesis [7] and the closure of ATP-sensitive K^+^ channels [8] cause plasma membrane depolarisation, Ca^2+^ influx via voltage-gated channels (L-type) [9], the further activation of mitochondrial metabolism [10], and secretory granule exocytosis [11]. In addition, ATP-sensitive K^+^ channel-independent mechanisms potentiate the actions of Ca^2+^ via mechanisms that are presently poorly understood [12]. The signalling pathways controlling glucagon secretion are less well defined and may involve a more dominant role for neural and hormonal control [13], as well as the cell-intrinsic actions of glucose. Enhanced ATP synthesis [14], ATP-sensitive K^+^ channel closure and the opening of T-type Ca^2+^ channels [15], and ATP-dependent effects on intracellular Ca^2+^ stores may also be involved [16].

Fuel-sensing kinases such as AMP-activated protein kinase [17] have previously been implicated in the control of both insulin [18, 19] and glucagon secretion [20, 21]. Originally cloned in 2001 by Hofer and colleagues [22] and, separately, by Rutter and colleagues [23], Per-Arnt-Sim (PAS) domain-containing protein kinase (PASK, also known as PASKIN) is a member of the nutrient-regulated protein-serine kinase family and the only PAS domain-containing member of this group expressed in mammalian cells [24]. In *Saccharomyces cerevisiae* there are two PASK orthologues: *Psk1* and *Psk2*. Under cell wall stress conditions these enzymes phosphorylate the metabolic enzyme uridine diphosphate-glucose pyrophosphorylase to mobilise glucose [25]. Importantly, the PAS domain of the mammalian enzyme is potentially targetable by small molecules and as such may offer an attractive target for therapeutic intervention in some forms of diabetes [24].

Implying a potentially important role for PASK in regulated insulin secretion, we showed in 2004 that PASK is required in clonal pancreatic beta cells for normal proinsulin gene expression [26]. Subsequently, Rutter and colleagues [27] demonstrated a role for PASK in mammalian glucose homeostasis by showing that mice deleted globally for the enzyme displayed improved insulin sensitivity but impaired insulin secretion in vivo and from isolated islets. We have also shown that activating mutations in the human *PASK* gene modulate insulin secretion [28]. However, subsequent studies [29] revealed little effect of global *Pask* deletion in mice on in vivo insulin secretion, but a profound effect on total pancreatic insulin content, consistent with a role for PASK in insulin gene expression [26, 29, 30] and insulin protein stability [31]. Glucagon release was enhanced in this model [29].

The impact of tissue-selective deletion of conditional *Pask* alleles has not hitherto been explored, restricting our understanding of the role of the kinase in glucose homeostasis in vivo. Here, we describe the generation of floxed alleles of the kinase and explore the impact of its deletion from pancreatic beta or alpha cells. Whereas fasting and fed insulin levels and glucose-stimulated insulin secretion were not affected by *Pask* deletion under most conditions, beta cell mass was significantly (36.5%) reduced in beta cell-selective null animals (*Pask*BKO). On the other hand, alpha cell-selective deletion (*Pask*AKO) led to impaired glucagon secretion in vitro and in vivo. These findings demonstrate the importance of PASK for the normal development and function of the beta and alpha cell, respectively.

## Methods

### Generation of *Pask*BKO and *Pask*AKO mice

Mice carrying conditional null alleles of *Pask* (*Pask*^*fl*/*fl*^) were generated by insertion of loxP sites flanking exon 11 and exon 15, which encompass the Ser/Thr kinase domain, through homologous recombination (see electronic supplementary material [ESM] Methods, ESM Fig. 1a–c).

*Pask*^*fl*/*fl*^ mice were crossed with animals expressing *Cre* recombinase under the control of the insulin 1 promoter (*Ins1*^Cre^ mice [32]) or with mice expressing *Cre* under the control of the glucagon promoter (*Ppg*^Cre^ mice [33]) to achieve deletion in pancreatic beta cells (*Pask*BKO) or in pancreatic alpha cells (*Pask*AKO). *Pask*BKO mice were born at the expected Mendelian ratios. *Pask*AKO mice were born at the expected Mendelian ratios only if assuming that the *Cre* transgene had co-localised on the same chromosome (chromosome 1) as the *Pask* floxed gene. Genotyping was performed by PCR using DNA from ear biopsies. Ablation of *Pask* gene expression from pancreatic islets was assessed by quantitative real-time PCR (qPCR) on cDNA reverse-transcribed from islet RNA, as described below. All mouse strains were maintained on a C57BL/6 background. Mice with global deletion of *Pask* [34] were gifts from R. Wenger (University of Zürich, Zürich, Switzerland).

### Mouse maintenance and diet

Animals were housed in groups of two to five per individually ventilated cage in a pathogen-free facility with a 12 h light/dark cycle and were fed ad libitum with a standard mouse chow diet or a high-fat diet (60% wt/wt fat content; Research Diets, New Brunswick, NJ, USA). Where indicated, 8-week-old mice were transferred on to a high-fat diet for a period of 12 weeks. All in vivo procedures described were performed at the Imperial College Central Biomedical Service and approved by the UK Home Office according to the UK Animals (Scientific Procedures) Act 1986 (PPL 70/7349 and PPL 70/7179).

In vivo physiology (IPGTT, in vivo glucose-stimulated insulin secretion, insulin tolerance tests, plasma glucagon, hyperinsulinaemic–hypoglycaemic clamps), RNA extraction and qPCR, islet isolation and hormone secretion, and beta and alpha cell mass measurements are described in ESM Methods. Blood was collected at indicated time points and plasma insulin was measured by ELISA (Mercodia, Uppsala, Sweden). Total cellular RNA was extracted from mouse islets or other tissues and converted to cDNA for qPCR. Alpha and beta cell mass was assessed in pancreases from 20-week-old mice. Anti-PASK antibody (PA5-29309; Pierce, Rockford, IL, USA) was used in immunohistochemical analysis. Experimenters were blinded to the group assignment for assessment of islet cell mass. Samples were not randomised. No data, samples or animals were excluded.

### Statistical analysis

Data are expressed as means ± SEM. Significance was tested by unpaired or paired two-sample Student’s *t* tests using Excel, or by ANOVA using GraphPad 4.0 (La Jolla, CA, USA). A value of *p* < 0.05 was considered significant.

## Results

### Generation of mice deleted for *PASK* selectively in the pancreatic beta or alpha cell

**Fig. 1:**
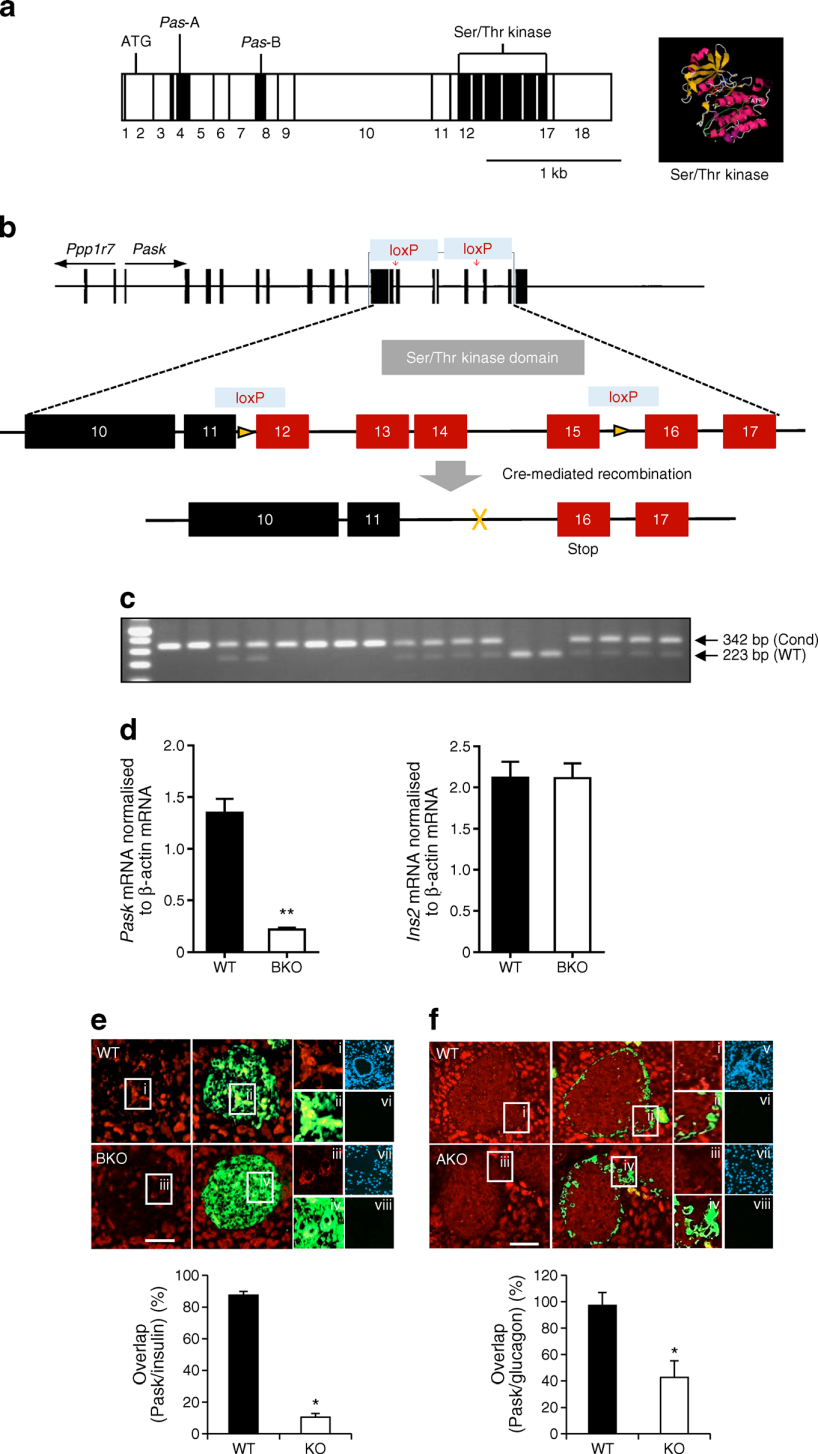
Generation of conditional *Pask* alleles and deletion in beta or alpha cells. (**a**) Knockout (KO) strategy. Generation of beta (*Pask*BKO) and alpha (*Pask*AKO) cell-specific *Pask* KO mice by Cre-mediated excision of exons from 12 to 15 encoding the PASK Ser/Thr kinase domain. Human PASK cDNA (modified from [22]) with the Ser/Thr kinase domain encoding exons circled in purple and enlarged in the black box—a view from the back of PASK kinase domain (amino acids 977–1300) crystal structure (PBD code 3DLS) with the bound ADP molecule in evidence [43]. (**b**) Mouse *Pask* gene structure (modified from [22]) and the location of the loxP sites. (**c**) Example of a genotyping gel indicating the presence of WT and recombined (Cond) alleles. (**d**) *Pask* and *Ins2* gene expression measured by qPCR in *Pask*BKO (BKO) and control (WT) mouse islets. Immunohistochemical analysis of pancreatic sections from *Pask*BKO (**e**) and *Pask*AKO (**f**) mice and controls, with PASK revealed using Alexa 568 (red), and insulin or glucagon revealed using Alexa 488 (green). Insets i–iv are magnified areas, as shown in the main images. Insets v–viii are example images of no-primary-antibody controls for each of the genotypes, with insets v and vii showing the DAPI staining for nuclei; scale bars, 100 μm. PASK fluorescence was displayed in 87.6 ± 1.5% of insulin-positive cells in WT islets vs 10.5 ± 2.3% in *Pask*BKO islets; *n* = 7 mice per genotype for both *Pask*BKO and *Pask*AKO mice; *p < 0.05 and **p < 0.01 by Student’s *t* test. Data were obtained by counting pixel overlay from ten pancreatic slices (typically counting approximately ten islets per slice) per mouse using Fiji (see ESM Methods)

Breeding of mice with floxed *Pask* alleles with animals expressing *Cre* recombinase under the control of the *Ins1* [32] or *Ppg* gene promoter [33] was predicted to lead to recombination selectively in pancreatic islet beta (*Pask*BKO) or alpha (*Pask*AKO) cells, respectively. Correspondingly, *Pask* mRNA levels, assessed by qPCR, were reduced by >75% in *Pask*BKO mouse islets vs controls (Fig. 1d, left), with concomitant reduction in beta cell PASK protein content, as assessed by immunohistochemistry (Fig. 1e). No change in *Ins2* mRNA levels (Fig. 1d, right) was detected in the same islets. By contrast, differences in *Pask* mRNA could not be detected between *Pask*AKO and wild-type (WT) islets (not shown), likely reflecting the low abundance of alpha cells in rodent islets (~20% of all cells) [35] and expected 20–50% deletion with the *Ppg*^Cre^ used here [21]. Correspondingly, in *Pask*AKO islets, immunohistochemical analysis revealed a 42.6 ± 17.5% overlap of the signal from anti-PASK antibody with that from glucagon antibody, whereas this value was close to 100% in WT islets (Fig. 1f).

### Pancreatic *Pask*BKO slightly impairs glucose homeostasis after high-fat feeding

**Fig. 2:**
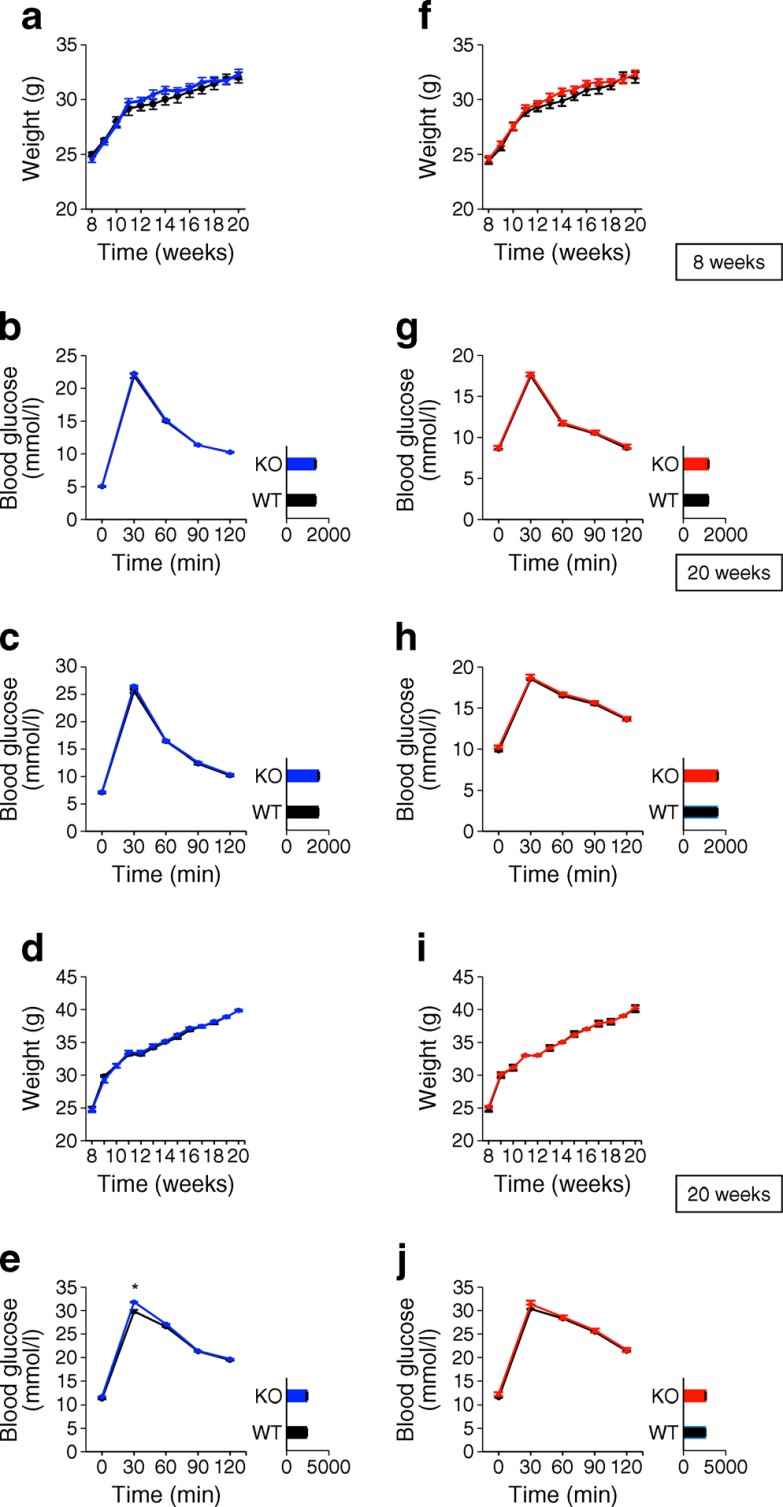
*Pask*BKO, but not *Pask*AKO, mice on a high-fat diet display mild glucose intolerance after i.p. glucose injection. Body weight (**a**, **f**) and IPGTT for *Pask*BKO (blue), *Pask*AKO (red) and WT (black) male mice on a normal chow diet at (**b**, **g**) 8 weeks, and at (**c**, **h**) 20 weeks. Body weight (**d**, **i**) and IPGTT for *Pask*BKO (blue), *Pask*AKO (red) and WT (black) male mice on a high-fat diet at (**e**, **j**) 20 weeks; *n* = 7 for each genotype; *p < 0.05 by Student’s *t* test with Bonferroni correction; AUCs (insets) given in arbitrary units

**Fig. 3:**
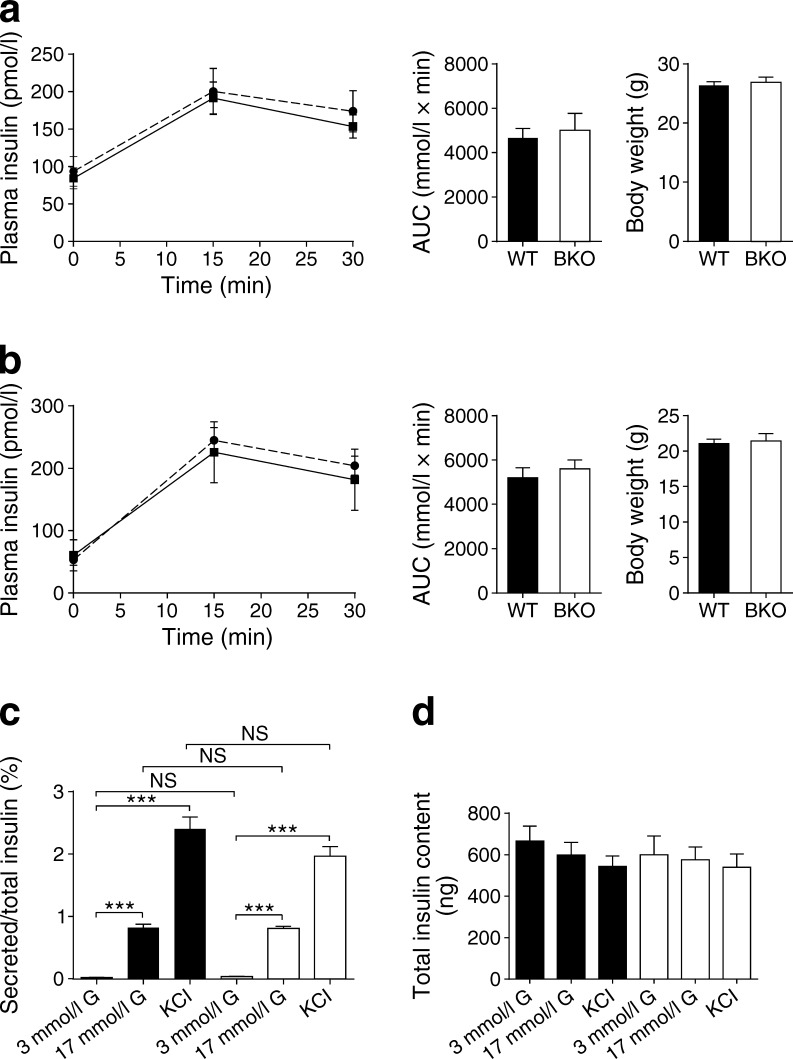
*Pask*BKO mice display normal glucose-stimulated insulin secretion both in vivo after intraperitoneal glucose injection and in vitro from isolated islets. (**a**, **b**) In vivo glucose-stimulated insulin secretion: 13- to 15-week-old mice [*Pask*BKO (BKO), dashed black line on line graph or white column on histogram, male (**a**) *n* = 4, female (**b**) *n* = 5; WT, solid black line on line graph or black bar on histogram, male (**a**) *n* = 5, female (**b**) *n* = 6] were injected with 3 g glucose/kg body weight. (**c**) In vitro glucose-stimulated insulin secretion from islets isolated from *Pask*BKO (white columns, *n* = 4) and WT (black columns, *n* = 4) mice at 3 mmol/l and 17 mmol/l glucose (G) or 3 mmol/l glucose plus 20 mmol/l KCl. Statistical comparisons were made using one-way ANOVA; ***p < 0.001

Measured in male mice at 8 and 20 weeks of age, *Pask*BKO animals on a normal chow diet displayed normal weight gain (Fig. 2a) and normal glucose homeostasis during IPGTT compared with WT littermates (Fig. 2b, c). Both in vivo (1 g/kg; Fig. 3a, b) and in vitro (Fig. 3c) glucose-stimulated insulin secretion were unaffected by the deletion of *Pask*.

In order to provide a metabolic stress on the beta cell, where effects of *Pask* deletion might become apparent, we challenged *Pask*BKO mice with a high-fat diet for 12 weeks. Weight gain between WT and null mice was not significantly different (Fig. 2d). *Pask*BKO animals exposed to a high-fat diet had an elevated glucose peak during IPGTT compared with WT littermates (Fig. 2e), although no significant differences in AUC for glucose excursions (Fig. 2e) or plasma insulin (Fig. 3a, b) were observed.

### Pancreatic beta cell mass is lowered in *Pask*BKO mice and global *Pask*-null mice

**Fig. 4:**
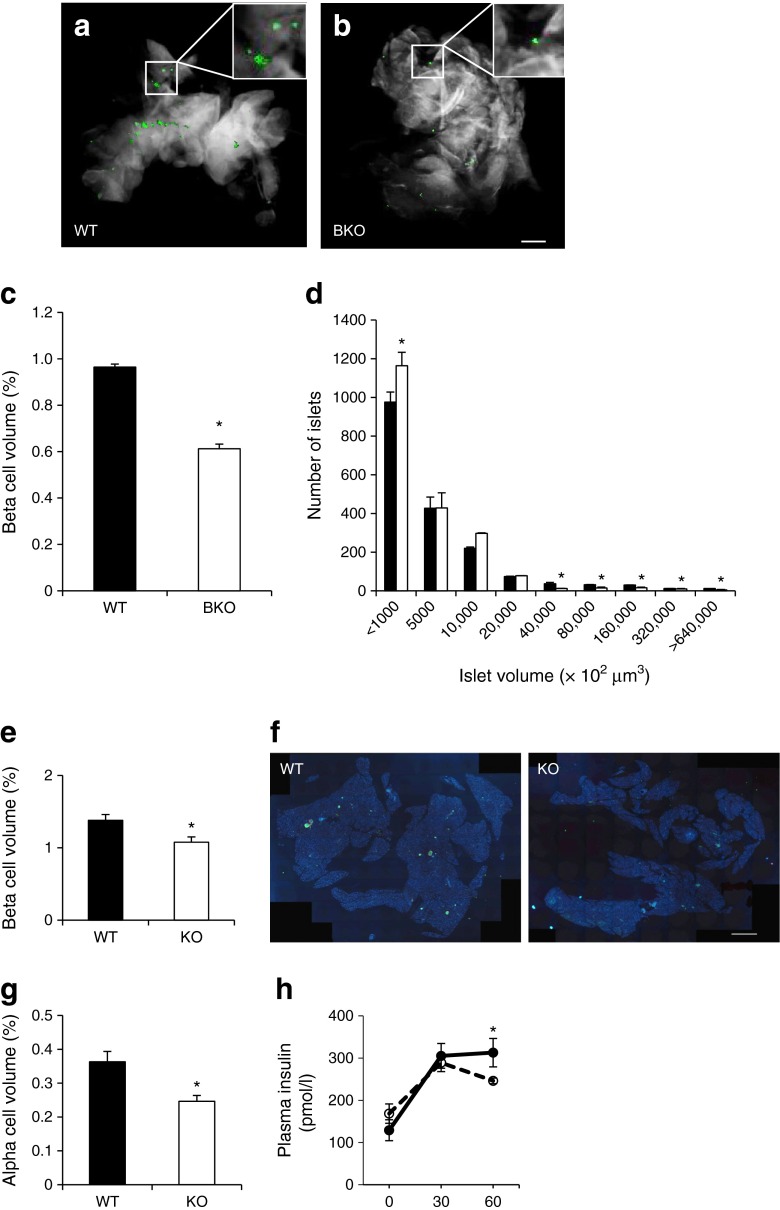
Beta and alpha cell volume and plasma insulin levels in *Pask*BKO and global *Pask*-null mice. Beta cell volume was measured by optical projection tomography, as described in the Methods section. Representative images of pancreases from WT (**a**) and *Pask*BKO (**b**) mice are shown, with insulin staining in green; scale bar, 500 μm. Quantification of beta cell relative to total pancreas volume (**c**) and size distribution of islets (**d**); *Pask*BKO in white, WT in black. (**e**, **g**) Beta and alpha cell mass and (**h**) plasma insulin levels after an IPGTT in global *Pask*-null mice (KO, white columns or dotted line; WT in black columns or solid line) were assessed by wide-field microscopy and following immunohistochemistry, respectively. (**f**) Representative images of pancreatic sections from WT and global *Pask*-null mice; scale bar, 1000 μm. Data were from at least four mice per genotype, aged 20 weeks; *p ≤ 0.05 by Student’s *t* test with Bonferroni correction for multiple tests

**Fig. 5:**
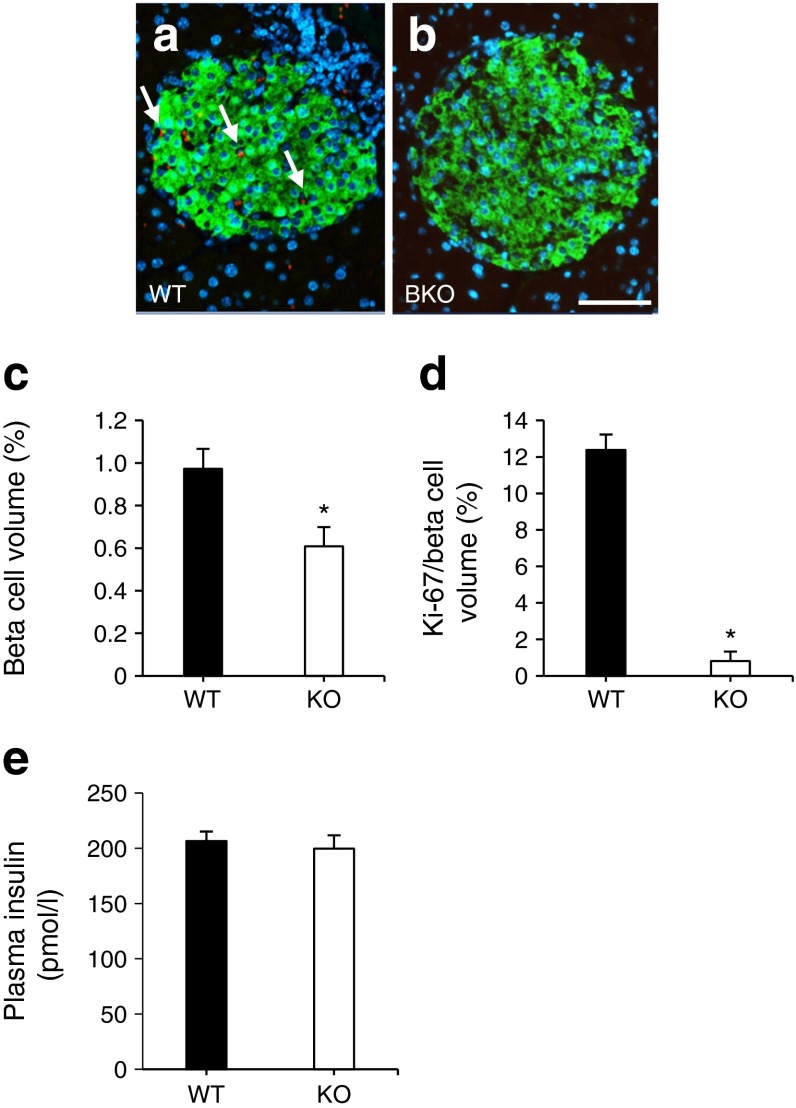
*Pask*BKO islets from mice that have been exposed to a high-fat diet have lowered beta cell mass and Ki-67 immunoreactivity. Ki-67 levels (revealed using Alexa 568, red) and insulin (revealed using Alexa 488, green) were assessed by immunohistochemistry, as described in the Methods section. Blue indicates DAPI staining for nuclei. Representative images of islets from WT (**a**) and *Pask*BKO (**b**) islets and quantification of beta cell mass (**c**) are shown. Ki-67 expression is shown as a percentage of beta cell volume (**d**). Plasma insulin content is shown in (**e**). Arrows indicate Ki-67 staining (**a**); scale bar, 50 μm; *p ≤ 0.05 by Student’s *t* test with Bonferroni correction

The above observations suggested that beta cell function or mass may be affected by *Pask* deletion. Since a beta cell phenotype may be masked or compensated for in vivo, we further investigated this in detail by performing in vitro experiments and by quantifying islet mass. To explore the latter possibility, optical projection tomography or immunohistochemistry were undertaken and revealed a 36.5 ± 1.2% (Fig. 4a–d) and a 38 ± 1.93% (Fig. 4e) decrease in beta cell mass in *Pask*BKO and global *Pask*-null mice, respectively. In *Pask*BKO animals, this reflected both increases in the number of smaller, and decreases in the number of larger, islets (Fig. 4d). Interestingly, after maintenance on a high-fat diet, we observed a similar decrease in beta cell mass (Fig. 5c), as well as a near-complete elimination of Ki-67 staining, in beta cells of *Pask*BKO vs WT islets (Fig. 5a, b and d), indicating impaired proliferation under these conditions. Fed plasma insulin levels were, nonetheless, unchanged in this model (Fig. 5e).

### Pancreatic alpha cell mass is lowered in global *Pask*-null mice but increased in *Pask*AKO mice

**Fig. 6:**
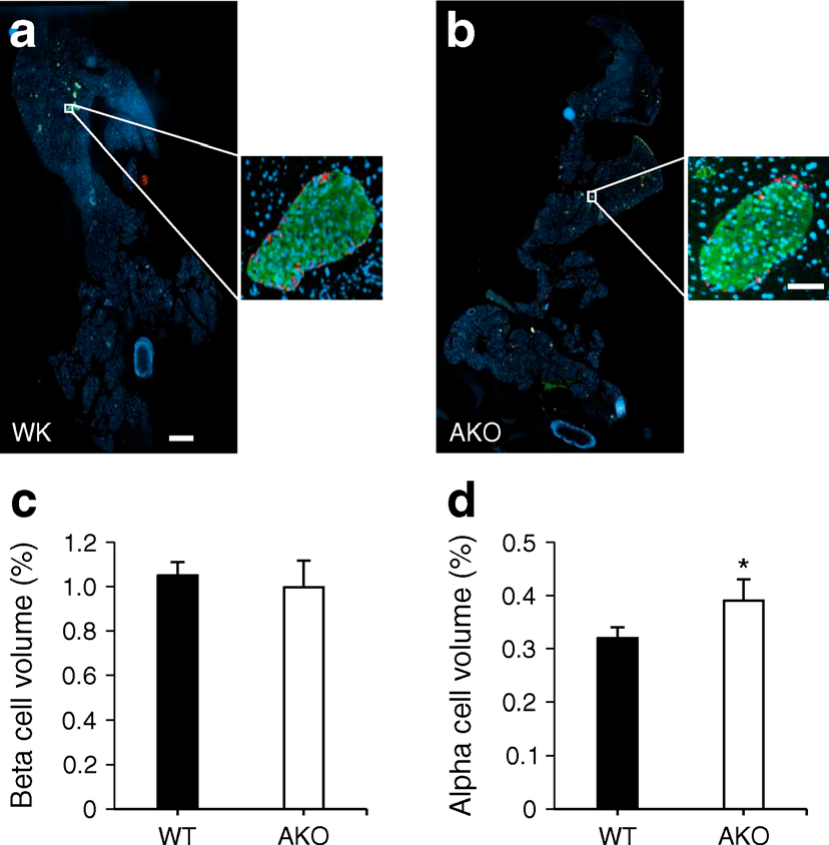
*Pask*AKO mice display increased alpha cell mass. Immunohistochemical analysis of pancreases from male *Pask*AKO mice at 20 weeks of age was performed as described in the Methods section. Insulin and glucagon were revealed using Alexa 488 (green) and 568 (red), respectively, with nuclei labelled using DAPI (blue). (**a**, **b**) Typical images from pancreatic sections from WT littermate controls (black bars) and *Pask*AKO (white bars) mice, respectively. Scale bar, 500 μm; insets show enlarged images of a typical islet; inset scale bar, 50 μm. (**c**, **d**) Per cent beta and alpha cell volume determined using Fiji. Data are from seven mice; *p < 0.05 using Student’s *t* test with Bonferroni correction

Immunohistochemical analysis revealed a 47.4 ± 0.7% decrease in alpha cell mass in global *Pask*-null mice (Fig. 4g). Whilst no significant changes in beta cell mass were observed in *Pask*AKO mice (Fig. 6a–c), a 21.9 ± 4.6% increase in alpha cell mass was detected in *Pask*AKO vs WT mice (Fig. 6d).

### Abnormal counterregulatory responses and glucagon secretion in *Pask*AKO mice

Male *Pask*AKO mice on a normal chow diet or a high-fat diet showed no significant differences in weight gain or glucose tolerance between 8 and 20 weeks (Fig. 2f–j).

**Fig. 7:**
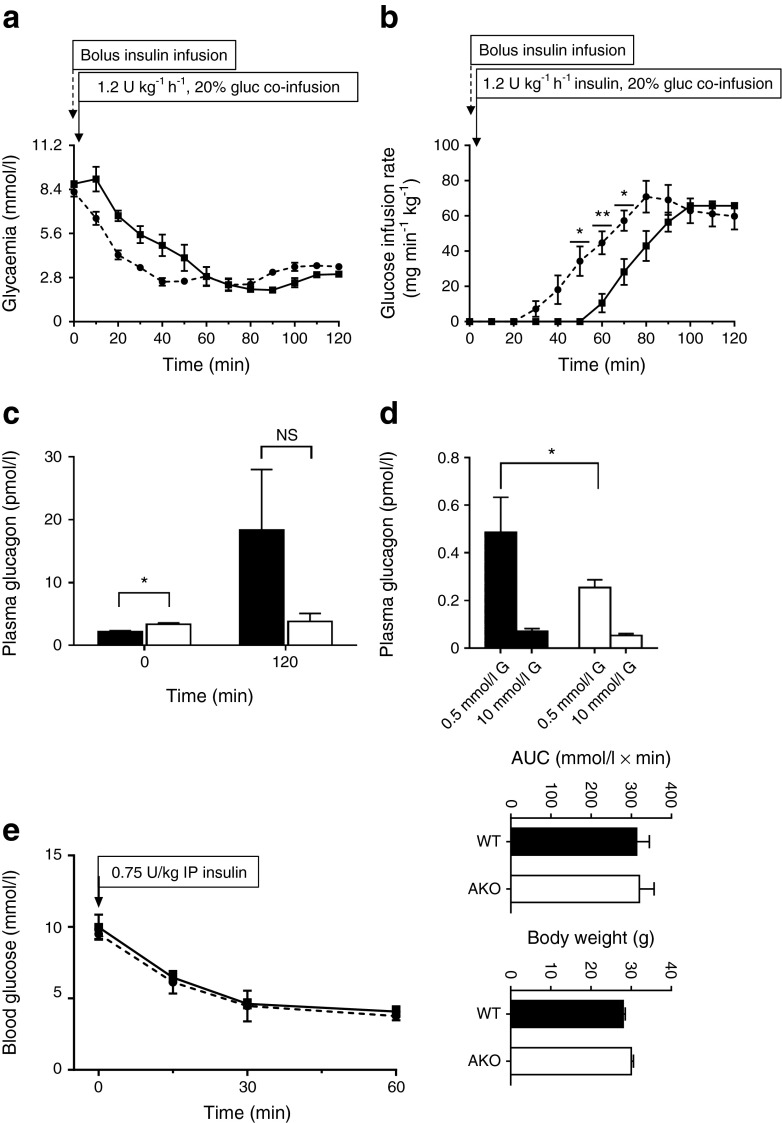
Hyperinsulinaemic–hypoglycaemic clamps in *Pask*AKO and WT mice, and release of glucagon from isolated islets. Blood glucose levels (**a**) and glucose infusion rate (**b**) measured in *Pask*AKO (dotted line) and WT littermates (solid line) during insulin (1.2 U kg^−1^ h^−1^) and glucose (gluc, 20%) co-infusion 5 min after bolus insulin infusion (0 min). The glucose infusion rate is indicated in (**b**). (**c**) Blood glucagon measurement before (0 min) and at the end (120 min) of the clamp. Data are expressed as means ± SEM; *n* = 3–5 mice per genotype. (**d**) Glucagon release from *Pask*AKO mouse and control islets in response to low (0.5 mmol/l) or high (10 mmol/l) glucose (G). (**e**) Insulin (0.75 U/kg, 0 min) tolerance test on 24-week-old male *Pask*AKO (*n* = 3; dotted line) or WT (*n* = 5; solid line) mice. *p < 0.05, **p < 0.01 by Student’s *t* test with Bonferroni correction

We performed hyperinsulinaemic–hypoglycaemic clamps to explore the impact of deleting *Pask* in the alpha cell on in vivo glucagon production. Strikingly, upon insulin infusion, the decrease in glycaemia was more rapid in *Pask*AKO animals vs WT mice, with the former reaching 0.5 g/l at around 40 min compared with 60 min in controls (Fig. 7a). Furthermore, during the time period 60–120 h, when glycaemia was similar in both groups (around 0.5 g/l), it was necessary to infuse more glucose in *Pask*AKO mice, confirming a likely lack of counterregulatory response in these mice (Fig. 7b). Although a small but significant increase in glucagon levels was apparent prior to the onset of the glucose clamps (Fig. 7c), glucagon release at 120 min displayed a strong tendency towards a lowering in the knockout mice (Fig. 7c). Similarly, glucagon secretion from isolated islets was significantly reduced in response to low glucose (Fig. 7d). By contrast, we observed no differences in insulin tolerance between mice of either genotype in vivo (Fig. 7e).

## Discussion

We provide here the first description of mice deleted highly selectively for *Pask* in the pancreatic alpha or beta cell.

We were able to demonstrate a robust lowering of *Pask* expression in islets in both *Pask*BKO and *Pask*AKO mice, reflecting the expected efficiency of the *Ins1*^Cre^ deleter strain (found to recombine in >94% of beta cells, with no significant off-target recombination in other cell types) [32, 36] and the *Ppg*^Cre^ strain (which recombines in 20–50% of alpha cells) [37].

### Role of PASK in the beta cell

Whilst insulin secretion from islets was previously shown to be defective in global *Pask*-null mice [27], our own studies with this model [29] indicated that the stimulation of insulin release was normal. However, *Ins2* and *Pdx1* gene expression were both substantially lower in knockout islets, and the pancreatic content of the hormone was sharply reduced [29]. In the present study using the *Ins1*^Cre^ line [36, 38] to inactivate *Pask* in the beta cell, we did not detect decreases in insulin mRNA measured per islet, but did observe a significant lowering of beta cell mass throughout the pancreas (Fig. 4c, d), which was paralleled by decreased Ki-67 immunoreactivity when measured after a high-fat diet (Fig. 5a–d). Furthermore, in vivo measurements of insulin revealed no significant decreases in plasma insulin following an IPGTT in *Pask*BKO mice when measured at 13–15 weeks (Fig. 3a, b), although there was a tendency towards lowered plasma insulin when measured between 16 and 20 weeks (data not shown). By contrast, global *Pask*-null mice displayed both decreases in beta cell mass (Fig. 4e) and in vivo insulin secretion (Fig. 4h).

The reason(s) for the modest effect of beta cell-specific elimination of PASK, in contrast to findings in the global *Pask*-null mouse here and in earlier reports [26, 29–31], is unclear. However, these results may suggest that changes in the global *Pask*-null mice reflect either (1) a requirement for PASK during the development of beta cell progenitors, and/or (2) the downstream consequences of *Pask* deletion elsewhere in the body, possibly including the brain [39]. With respect to the first, Semache and colleagues [31] have previously reported that PASK regulates the stability of pancreatic duodenal homeobox 1, a key regulator of the insulin gene [40], which might be expected to restrict beta cell development and may underlie an observed decrease of ~37% beta cell mass in the *Pask*BKO mouse found here. Of note, such a decrease is not expected to induce glucose dyshomeostasis, given that the destruction of 50% of beta cells is without effect in mice [41]. Thus, impaired glucose intolerance in *Pask*BKO mice after a high-fat diet is likely to reflect additional changes to pancreatic beta cell function and glucose sensing.

### Role of PASK in the alpha cell

Our previous findings [29] pointed to a role for PASK in the control of glucagon secretion. Here, we demonstrate this role in vivo after selective deletion from the alpha cell population. Unexpectedly, whilst we confirmed our previous finding of enhanced basal release of the hormone in global *Pask*-null mice (Fig. 7c), suggesting that PASK restricts glucagon release at normal levels of glycaemia, we now demonstrate that PASK is required for the stimulation of glucagon release during hypoglycaemia. Thus, glycaemia was lowered more rapidly in response to insulin infusion in *Pask*AKO mice than in controls, despite higher glucose infusion rates (Fig. 7). Of note, these changes were apparent despite deletion in 57.4% of alpha cells with the Cre deleter strain used here [21, 37]. Given the observed decrease in alpha cell mass of ~47% in the global *Pask*-null mouse (Fig. 4g), we hypothesised that decreases in alpha cell number may contribute to impaired glucagon production in *Pask*AKO mice. However, *Pask*AKO mice displayed an *increase* in alpha cell mass (Fig. 6d), indicating that alpha cell dysfunction is likely to be the major contributor to the phenotype displayed by *Pask*AKO mice.

The reasons for the differences in the impact on alpha cell mass of global vs cell type-specific *Pask* deletion are unclear. Furthermore, the above findings are in contrast to earlier observations with global *Pask*-null animals [29], in which fasting glucagon levels were higher in *Pask*-null mice than in WT mice, and the ability of high glucose to suppress glucagon secretion was impaired in islets from the former. What may underlie these differences? Again, actions of the kinase at extrapancreatic sites (such as liver, adipocytes and brain) are one possibility, whilst a role for PASK in restricting the production of alpha cell progenitors, mooted above, may provide an alternative explanation. In support of this idea, alpha cell mass was decreased in global *Pask*-null mice (Fig. 4g). Alpha cell function is also regulated through insulin secretion by neighbouring beta cells and it is therefore conceivable that the effects on glucagon secretion observed in the whole-body knockout mice may reflect a combination of defects in all pancreatic cell types in this model that are not replicated in the present cell type-specific models. It is currently unknown, for example, whether PASK has any function in pancreatic delta cells, although somatostatin is a regulator of both alpha and beta cell function [42]. Nevertheless, the present results demonstrate a clear and cell-autonomous role for PASK in the acute control of glucagon secretion (Fig. 7c, d) and beta cell mass (Fig. 5). The precise molecular mechanisms through which the kinase acts will need further exploration, and possibly the generation of mice bearing selectively-labelled alpha cells to permit studies at the single cell level [37].

### Conclusions

The present findings indicate that PASK plays critical roles in the biology of both pancreatic beta and alpha cells in vivo. However, we reveal striking differences between the glycaemic phenotype of animals deleted globally for the kinase vs the cell type-specific knockouts, demonstrating likely important roles for tissue interactions or actions at early developmental stages.

## Electronic supplementary material

Below is the link to the electronic supplementary material.

ESM (PDF 255 kb)

## Abbreviations

PAS: Per-Arnt-Sim
PASK: Per-Arnt-Sim kinase
*Pask*AKO: Alpha cell-selective deletion of *Pask*
*Pask*BKO: Beta cell-selective deletion of *Pask*
qPCR: Quantitative real-time PCR
WT: Wild-type

## References

[1] Guariguata L, Whiting DR, Hambleton I, Beagley J, Linnenkamp U, Shaw JE (2014). Global estimates of diabetes prevalence for 2013 and projections for 2035. Diabetes Res Clin Pract.

[2] Kahn SE, Zraika S, Utzschneider KM, Hull RL (2009). The beta cell lesion in type 2 diabetes: there has to be a primary functional abnormality. Diabetologia.

[3] Parker VE, Savage DB, O'Rahilly S, Semple RK (2011). Mechanistic insights into insulin resistance in the genetic era. Diabet Med.

[4] Thorens B, Sarkar HK, Kaback HR, Lodish HF (1988). Cloning and functional expression in bacteria of a novel glucose transporter present in liver intestine kidney and B-pancreatic islet cells. Cell.

[5] Rutter GA, Pullen TJ, Hodson DJ, Martinez-Sanchez A (2015). Pancreatic beta cell identity, glucose sensing and the control of insulin secretion. Biochem J.

[6] Hutton JC, Sener A, Herchuelz A (1980). Similarities in the stimulus-secretion coupling mechanisms of glucose- and 2-keto acid-induced insulin release. Endocrinology.

[7] Tarasov AI, Griffiths EJ, Rutter GA (2012). Regulation of ATP production by mitochondrial Ca^2+^. Cell Calcium.

[8] Ashcroft FM, Rorsman P (2013). K_ATP_ channels and islet hormone secretion: new insights and controversies. Nat Rev Endocrinol.

[9] Rutter GA, Theler J-M, Murta M, Wollheim CB, Pozzan T, Rizzuto R (1993). Stimulated Ca^2+^ influx raises mitochondrial free Ca^2+^ to supramicromolar levels in a pancreatic β-cell line: possible role in glucose and agonist-induced insulin secretion. J Biol Chem.

[10] Rutter GA, Pralong W-F, Wollheim CB (1992). Regulation of mitochondrial glycerol phosphate dehydrogenase by Ca^2+^ within electropermeabilized insulin secreting cells (INS-1). Biochim Biophys Acta.

[11] Rutter GA (2004). Visualising insulin secretion. The Minkowski lecture 2004. Diabetologia.

[12] Henquin JC (2009). Regulation of insulin secretion: a matter of phase control and amplitude modulation. Diabetologia.

[13] Tarussio D, Metref S, Seyer P (2014). Nervous glucose sensing regulates postnatal beta cell proliferation and glucose homeostasis. J Clin Investig.

[14] Ravier MA, Rutter GA (2005). Glucose or insulin, but not zinc ions, inhibit glucagon secretion from mouse pancreatic α-cells. Diabetes.

[15] Gopel SO, Kanno T, Barg S, Weng X, Gromada J, Rorsman P (2000). Regulation of glucagon release in mouse-cells by KATP channels and inactivation of TTX-sensitive Na+ channels. J Physiol.

[16] Liu YJ, Vieira E, Gylfe E (2004). A store-operated mechanism determines the activity of the electrically excitable glucagon-secreting pancreatic alpha-cell. Cell Calcium.

[17] Rutter GA, Leclerc I (2009). The AMP-regulated kinase family: enigmatic targets for diabetes therapy. Mol Cell Endocrinol.

[18] da Silva Xavier G, Leclerc I, Varadi A, Tsuboi T, Moule SK, Rutter GA (2003). Role for AMP-activated protein kinase in glucose-stimulated insulin secretion and preproinsulin gene expression. Biochem J.

[19] da Silva Xavier G, Leclerc I, Salt IP (2000). Role of AMP-activated protein kinase in the regulation by glucose of islet beta-cell gene expression. Proc Natl Acad Sci U S A.

[20] Kuznetsov JN, Leclerc GJ, Leclerc GM, Barredo JC (2011). AMPK and Akt determine apoptotic cell death following perturbations of one-carbon metabolism by regulating ER stress in acute lymphoblastic leukemia. Mol Cancer Ther.

[21] Sun G, da Silva Xavier G, Gorman T (2015). LKB1 and AMPKalpha1 are required in pancreatic alpha cells for the normal regulation of glucagon secretion and responses to hypoglycemia. Mol Metab.

[22] Hofer T, Spielmann P, Stengel P (2001). Mammalian PASKIN, a PAS-serine/threonine kinase related to bacterial oxygen sensors. Biochem Biophys Res Commun.

[23] Rutter J, Michnoff CH, Harper SM, Gardner KH, McKnight SL (2001). PAS kinase: an evolutionarily conserved PAS domain-regulated serine/threonine kinase. Proc Natl Acad Sci U S A.

[24] Sabatini PV, Lynn FC (2015). All-encomPASsing regulation of beta-cells: PAS domain proteins in beta-cell dysfunction and diabetes. Trends Endocrinol Metab.

[25] Grose JH, Smith TL, Sabic H, Rutter J (2007). Yeast PAS kinase coordinates glucose partitioning in response to metabolic and cell integrity signaling. EMBO J.

[26] da Silva Xavier G, Rutter J, Rutter GA (2004). Involvement of Per-Arnt-Sim (PAS) kinase in the stimulation of preproinsulin and pancreatic duodenum homeobox 1 gene expression by glucose. Proc Natl Acad Sci U S A.

[27] Hao HX, Cardon CM, Swiatek W (2007). PAS kinase is required for normal cellular energy balance. Proc Natl Acad Sci U S A.

[28] Semplici F, Vaxillaire M, Fogarty S (2011). A human mutation within the per-ARNT-sim (PAS) domain-containing protein kinase (PASK) causes basal insulin hypersecretion. J Biol Chem.

[29] da Silva Xavier G, Farhan H, Kim H (2011). Per-arnt-sim (PAS) domain-containing protein kinase is downregulated in human islets in type 2 diabetes and regulates glucagon secretion. Diabetologia.

[30] Fontes G, Semache M, Hagman DK (2009). Involvement of Per-Arnt-Sim kinase and extracellular-regulated kinases-1/2 in palmitate inhibition of insulin gene expression in pancreatic beta-cells. Diabetes.

[31] Semache M, Zarrouki B, Fontes G (2013). Per-Arnt-Sim kinase regulates pancreatic duodenal homeobox-1 protein stability via phosphorylation of glycogen synthase kinase 3beta in pancreatic beta-cells. J Biol Chem.

[32] Thorens B, Tarussio D, Maestro MA, Rovira M, Heikkila E, Ferrer J (2015). Ins1 knock-in mice for beta cell-specific gene recombination. Diabetologia.

[33] Herrera PL (2000). Adult insulin- and glucagon-producing cells differentiate from two independent cell lineages. Development.

[34] Katschinski DM, Marti HH, Wagner KF (2003). Targeted disruption of the mouse PAS domain serine/threonine kinase PASKIN. Mol Cell Biol.

[35] Elayat AA, el-Naggar MM, Tahir M (1995). An immunocytochemical and morphometric study of the rat pancreatic islets. J Anat.

[36] Kone M, Pullen TJ, Sun G (2014). LKB1 and AMPK differentially regulate pancreatic beta-cell identity. FASEB J.

[37] Solomou A, Meur G, Bellomo E (2015). The zinc transporter Slc30a8/ZnT8 is required in a subpopulation of pancreatic α-cells for hypoglycemia-induced glucagon secretion. J Biol Chem.

[38] Patel D, Ythier D, Brozzi F, Eizirik DL, Thorens B (2015). Clic4, a novel protein that sensitizes β-cells to apoptosis. Mol Metab.

[39] Hurtado-Carneiro V, Roncero I, Egger SS (2014). PAS kinase is a nutrient and energy sensor in hypothalamic areas required for the normal function of AMPK and mTOR/S6K1. Mol Neurobiol.

[40] Melloul D, Marshak S, Cerasi E (2002). Regulation of insulin gene transcription. Diabetologia.

[41] Vinet L, Lamprianou S, Babic A (2015). Targeting GLP-1 receptors for repeated magnetic resonance imaging differentiates graded losses of pancreatic beta cells in mice. Diabetologia.

[42] Hauge-Evans AC, King AJ, Carmignac D (2009). Somatostatin secreted by islet delta-cells fulfills multiple roles as a paracrine regulator of islet function. Diabetes.

[43] Kikani CK, Antonysamy SA, Bonanno JB (2010). Structural bases of PAS domain-regulated kinase (PASK) activation in the absence of activation loop phosphorylation. J Biol Chem.

