# Supplementary material for: Cell type-specific deletion in mice reveals roles for PAS kinase in insulin and glucagon production
Source: Diabetologia. 2016 Jun 24;59:1938–47. doi: 10.1007/s00125-016-4025-1 (PMC4969360; doi:10.1007/s00125-016-4025-1)
Supplement: Supplementary file 1 — (PDF 255 kb) [file 125_2016_4025_MOESM1_ESM.pdf]

SUPPLEMENTARY METHODS

Generation of Pask cKO mice.

A conditional knock-out strategy, flanking exons 12 and 15 with LoxP sites, was used to target the *Pask* locus in order to generate mice carrying conditional knock-out alleles at this site. The targeting vector was built using homologous recombination in bacteria BHR [1]. A C57 mouse bacterial artificial chromosome (BAC) served as template for the extraction of homology arms of the targeting vector (See Table). The 18 kb targeting vector, *Pask cKOTV*, contained a floxed neomycin phosphotransferase, Neo, selectable marker cassette, inserted 204 bp downstream of exon 11 and 200 bp upstream of exon 12. After linearization, the targeting construct was electroporated into AZX1, a C57Bl/6JOlaHsd derived ES cell line. PCR screens and Southern blot analyses (not shown) revealed that 4 out of 58 G418-resistant clones had undergone the desired homologous recombination. One of these clones was expanded and injected into Balb/cOlaHsd blastocysts to generate chimeric mice. Chimeric males were bred to C57Bl/6JOlaHsd females and black-coated offspring were genotyped on both sides of the homology arms for correct integration into the *Pask* locus. A forward primer (F1) in intron 9 outside the short arm homology combined with a reverse Neo-specific primer (R1) giving a PCR product of 3461 bp, and a LoxP specific primer F2 combined with a reverse primer outside the long arm, giving a PCR product of 6862 bp, was used for screening (Table 1).

F1:	TCGGTTGTTGTCATGCTAGGATTT
R1:	TAGTTGCCAGCCATCTGTTGTTT
F2:	GTTGCATGAGAAAGCAGACGCGTATAAC
R2:	TTAGGTGCCTTGTTCACCTGGATGTGTT
PASK 3' SA BHR	GGGGGATGCACACCACAACAACCTCATTAAGGCCTAGCCAAGGGGATATCGCTCATGAGACAATAACCCTG
PASK 5' SA BHR	ATGTAGGACTCTAAATGAATGTATACCTTGTACAGTGGGATGTAGATGCAGTCGACGGCGGTAATACGGTTATCCA
PASK 5' DF BHR	GGCCCCTCTGGCAGTGTCCAGCCATCAACCCAAACTGGCTTATGTCTAAAGCGGCCGCTCATGAGACAATAACCCTG
PASK 3' DF BHR	TGGGATGTGTTATCTCTGAGTGAGTGGTTCTGGGTTGCATGAGAAAGCAGACGCGTGGCGGTAATACGGTTATCCA
PASK LA 5' BHR	GGAACTGAAGCAGAGACCGTGGAGGAATCCTGTTTACTAGCTTGCTCAATCCTAGGACGTCTCATGAGACAATAACCCTG
PASK LA 3' BHR	TCAGTAGGTGCGCCCAGGCCAAACAATGCGGCTGGTCGGTGGTCAAAAAACTCGAGGCGGTAATACGGTTATCCA

Table 1: **Primer sequences used in the generation of the *Pask* floxed alleles**. Black: amplification of *Pask* sequences. Red: amplification of beta lactamase –ColE1 ori from pSK bluescript. Underlined: Restriction sites used for cloning of target vector.

***In vivo* physiology**

Intraperitoneal glucose tolerance test (IPGTT). Mice fasted for 16 h (with free access to water) were injected intraperitoneally with 1g glucose/kg, and blood glucose levels in tail vein blood measured with an automatic glucometer (Accuchek Compact Plus; Roche, Burgess Hill, UK). *In vivo glucose-stimulated insulin secretion*. Mice fasted for 16 h were intraperitoneally injected with 3g glucose/kg and tail vein blood collected into heparin-coated tubes (Sarstedt). Plasma was measured using an ultrasensitive mouse insulin ELISA kit (Mercodia, Uppsala, Sweden) or by radioimmunoassay (Linco/Millipore, Watford, U.K.). *Insulin tolerance tests.* Bovine insulin (0.75 U/kg; ActaRapid, NovoNordisk) was injected intraperitoneally into 16 h-fasted mice before glucose assay. *Plasma glucagon.* Blood (400-800 µl) was removed by cardiac puncture from either fed or overnight fasted mice terminally anaesthetized with Pentobarbital. Plasma was collected and centrifuged (2,000 g, 5

min.) in heparin-coated tubes (Microvette; Sarstedt, Leicester, UK) and plasma glucagon assessed by radioimmunoassay (Millipore/Linco). *Hyperinsulinemic/Hypoglycaemic clamps* were performed by perfusion of insulin and glucose solutions through a jugular catheter as described [2].

RNA extraction, reverse transcription and real time quantitative PCR (RT-qPCR)

Total cellular RNA from mouse islets or other tissues was obtained using TRIzol reagent (Invitrogen, Paisley, UK), and RNA was further purified against DNA contamination with a DNA-free kit (Applied biosystems, Warrington, UK). Total RNA (1.5–2 μg) was then reverse transcribed into cDNA with a high-capacity reverse transcription kit (Applied Biosystems) according to the manufacturer's instructions. qPCR was conducted using SYBR green (Applied Biosystems) on an ABI 7500 fast real time PCR machine.

Islet isolation and *in vitro* glucose stimulated insulin secretion (GSIS)

After sacrifice by cervical dislocation, islets were purified on histopaque gradients and hand-picked as described [3;4]. Islets were cultured in RPMI medium supplemented with 2 mM glutamine, 100 units/ml of penicillin, and 100 units/ml of streptomycin and 10% (v/v) heat-inactivated fetal bovine serum for 24–72 h. For *in vitro* measurement of glucose- and KCl-stimulated insulin secretion, 10 size-matched islets were preincubated for 1 h at 37 °C in Krebs-Ringer buffer (KRB, in mM: 130 NaCl, 3.6 KCl, 1.5 $CaCl_2$, 0.5 $MgSO_4$, 0.5 KH_2PO_4, 2 $NaHCO_3$, 10 HEPES, and 0.1% (w/v) BSA, pH 7.4, equilibrated for 20 min with O_2/CO_2 (95:5 (v/v)) at 37 °C) containing 3 mM glucose, with gentle agitation (120 rpm). Islets were further incubated for 2 h at 37 °C in KRB at 3 or 16.7 mM glucose. Secreted and total insulin levels were measured in triplicate by radioimmunoassay (Millipore, Watford, UK).

Glucagon secretion *in vitro*

Secretion from islets (15–20 per condition, size-matched) was measured in 0.5 mL KRB solution containing 0.5 or 10 mM glucose as described [2].

Optical projection tomography (OPT) and immunohistochemistry

Beta and alpha cell mass were assessed as previously described [5-7] on pancreata from 20 week old mice. Briefly, isolated pancreases were fixed in 10% buffered formalin and embedded in paraffin wax within 24 h of removal. Head-to-tail sections (5 µm lengthwise) were cut and incubated overnight at 37°C on superfrost slides. Slides were submerged sequentially in Histoclear (Sigma) followed by decreasing concentrations of industrial methylated spirits for removal of paraffin wax. Anti-PASK antibody (Pierce, PA5-29309) was used at 1:10 dilution following antigen retrieval (Vector antigen unmasking solution, Vector Biolabs). Images were captured on a Zeiss Axio Observer.Z1 Motorised Inverted Widefield Microscope fitted with a Hamamatsu Flash 4.0 Camera using Plan-Apochromat 20x/0.8 M27 air objective with Colibri.2 LED illumination. Data acquisition was controlled by Zeiss Zen Blue 2012 software configured at a bit depth of 16-bit and binning mode 2x2. Whole tissue tiled preview scans were obtained using an EC Plan-Neofluar 10x/0.3 Ph1 air objective with phase contrast. Excitation intensities and exposure times were kept constant for all images, including the negative controls. Image analysis was performed using Volocity (Invitrogen) and Fiji [8], as previously described [5-7].

Reference List

1. Datsenko KA, Wanner BL (2000) One-step inactivation of chromosomal genes in Escherichia coli K-12 using PCR products. Proc.Natl.Acad.Sci.U.S.A. 97: 6640-6645

2. Solomou A, Meur G, Bellomo E, et al (2015) The zinc transporter Slc30a8/ZnT8 is required in a subpopulation of pancreatic alpha cells for hypoglycemia-induced glucagon secretion. J.Biol.Chem. in press:

3. Sun G, Tarasov AI, McGinty JA, et al (2010) LKB1 deletion with the RIP.Cre-transgene modifies pancreatic {beta}-cell morphology and enhances insulin secretion in vivo. Am.J.Physiol Endocrinol.Metab. 298: E1261-E1273

4. Ravier MA, Rutter GA (2010) Isolation and culture of mouse pancreatic islets for ex vivo imaging studies with trappable or recombinant fluorescent probes. Methods Mol.Biol. 633: 171-184

5. da S, X, Farhan H, Kim H, et al (2011) Per-arnt-sim (PAS) domain-containing protein kinase is downregulated in human islets in type 2 diabetes and regulates glucagon secretion. Diabetologia. 54: 819-827

6. Alanentalo T, Asayesh A, Morrison H, et al (2007) Tomographic molecular imaging and 3D quantification within adult mouse organs. Nat.Methods. 4: 31-33

7. Mondragon A, Davidsson D, Kyriakoudi S, et al (2014) Divergent effects of liraglutide, exendin-4, and sitagliptin on beta-cell mass and indicators of pancreatitis in a mouse model of hyperglycaemia. PLoS.ONE. 9: e104873

8. Schindelin J, Arganda-Carreras I, Frise E, et al (2012) Fiji: an open-source platform for biological-image analysis. Nat.Methods. 9: 676-682

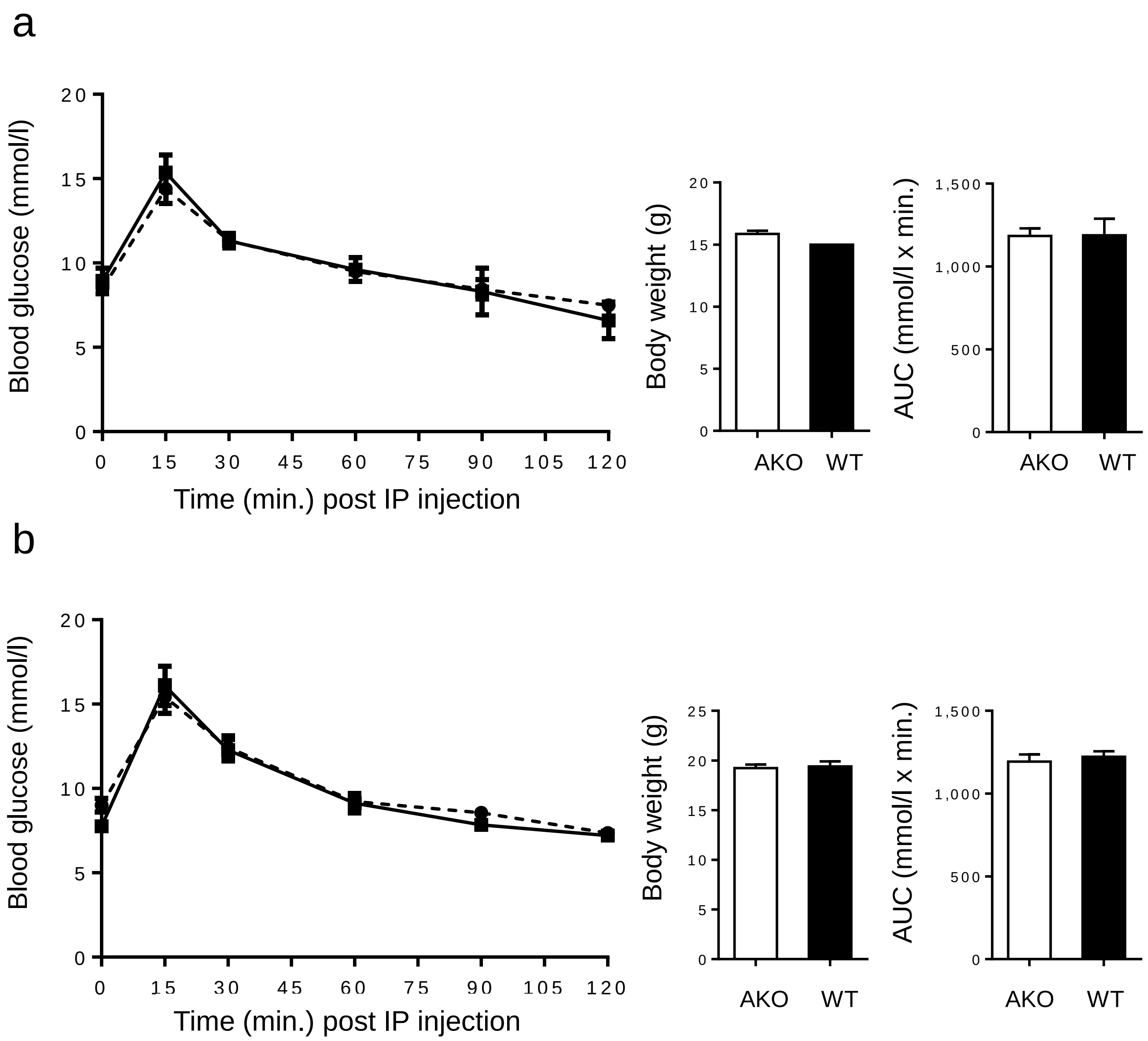

ESM Fig. 1: Female *Pask*AKO mice display normal glucose tolerance after intra-peritoneal glucose injection. (a) From left to right: IPGTT, body weight and area under the curve (AUC) for *Pask*AKO (circles, dashed line) and WT (squares, solid line) for 7-8 week old mice (*Pask*AKO, *n*=7, wild type, *n*=2). (b) as (a) but for animals at 11-12 weeks (*Pask*AKO, *n*=9, wild type, *n*=5).
